# Interventions to encourage discussion of end-of-life preferences between members of the general population and the people closest to them - a systematic literature review

**DOI:** 10.1186/1472-684X-12-40

**Published:** 2013-11-04

**Authors:** Katharine Abba, Paula Byrne, Siobhan Horton, Mari Lloyd-Williams

**Affiliations:** 1 University of Liverpool, Academic Palliative and Supportive Care Studies Group (APSCSG), Waterhouse Building, Block B 1st Floor, 1-5 Brownlow Street, Liverpool L69 3GL, UK; 2 St Luke’s (Cheshire) Hospice, Grosvenor House, Queensway, Winsford, Cheshire CW7 1BH, UK

**Keywords:** Public health, End of life, Palliative care, Health promotion

## Abstract

**Background:**

Discussing end of life preferences can be beneficial, and it is thought that the best time to have these conversations is usually when people are well. This review aims to establish current evidence for the effectiveness of community-based interventions to encourage people to consider, and to discuss with those closest to them, their preferences for end of life care or what they wish to happen after their death.

**Methods:**

A systematic literature review was undertaken. A systematic search was conducted using Scopus and Google, and academic experts were contacted. Studies were included if they evaluated interventions intended to encourage people to discuss their end of life preferences with those closest to them, or to address known barriers to these discussions. Reported outcomes had to relate to attitude or behaviour change in the target group, or target group perceptions of the intervention. Studies were excluded if the intervention targeted only people with a life-limiting illness, or intended specifically to facilitate communication of end of life preferences between patients and healthcare staff. Studies were systematically described and assessed for quality. There was no attempt to combine results of different studies.

**Results:**

The Scopus search identified 5,743 citations, and the Google search identified over 40,000, of which the first 40 pages were scanned. Five studies were included, four identified through the Scopus search and one from a book identified through Google. Three studies reported positive results, two were less positive. A peer education programme on end of life planning for older people, featuring small discussion workshops, was positively appraised by participants. An arts project bringing hospice users and school pupils together appeared to help normalise death for school pupils. A public information ‘roadshow’ engaged people using an informal questionnaire survey, facilitating conversations between people who participated together. Public lectures by physicians intending to promoting home death as a possibility were unsuccessful in changing attitudes at six months follow-up. A module on end of life planning delivered as part of ‘expert patient’ education programme on the management of chronic illness was not well received by participants.

**Conclusions:**

Available evidence highlights the importance of actively engaging people rather than passively providing information, and of ensuring an appropriate context for interventions. However, data are limited and there is a need for more research and for sharing of best practice.

## Background

In England and Wales, the annual death rate is around 1% [1]. In high income countries, most people die in old age; in England between 2008 and 2010, 66.7% of people who died were over the age of 75 and 36.2% were over the age 85 [2]. Three main end of life decline trajectories have been identified [3]; short period of decline typical of cancer (21%); long-term limitations with intermittent serious episodes typical of organ failure (21%); and prolonged dwindling typical of frail elderly people and people with dementia (20%). Additionally, 15% of people die suddenly and 24% die following other, varied trajectories.

While dying is not always associated with pain or suffering, people who are dying can suffer isolation, grief, anxiety and depression [4]. Carers of people who are dying, or those who are bereaved, may suffer from illnesses including depression [5] or complicated grief [6] and may feel isolated as people around them fail to offer support.

A recent systematic literature review revealed that people throughout the world share core ideals of a ‘good death’ [7], which include being free of pain and other symptoms, being with friends and family, not being a burden, being listened to, being able to decide about medical treatments [8] and being treated with respect. In some studies ‘having one’s affairs in order’ was highlighted as important, while religion or spirituality was important to some people [9-11]. Many people would like to be cared for at home during their final illness [12-14].

‘Having one’s affairs in order’ necessarily requires preparation which might also assist people to have other end of life care wishes met. People in the USA who discussed and recorded their preferences in the form of an Advance Directive (a legally-binding document of a person’s preferences for medical care in event that they become incapacitated) were more likely to receive end of life care in accordance with their wishes [15-17]. People with newly diagnosed life-threatening or life-limiting illness often find it difficult to talk about their end of life preferences at this time [18], and people who become ill suddenly may never have the opportunity. Such findings suggest probable benefits in discussing end of life preferences while well, before death seems close.

There is limited research evidence on the effects of talking about end of life preferences, and that which is available has focussed mainly on discussions between people with a life-limiting illness and their health care providers. A cohort study of people with advanced cancer in the USA found that those who discussed their end of life care preferences with their doctors suffered less in their last week of life and their relatives suffered less depression six months later [19]. A trial in Australia [20], which tested an intervention to facilitate advance care planning in elderly hospital inpatients, found the intervention improved end of life care and reduced stress, anxiety and depression in surviving relatives. A recently-published study from the USA, showed that people who discussed their end of life care preferences with their next of kin had a higher probability of receiving hospice care at the end of their life than those who had not undertaken any advanced care planning [21].

Despite the potential benefits, many people do not discuss or make plans for the end of their life. In a UK population survey in 2011, 69% had not talked to anybody about their wishes for the end of their life, including 55% of those aged over 65 [22]. A similar survey in Ireland revealed 78% had not discussed how they would like to be treated if they were dying [23].

Various possible reasons have been identified for this widespread lack of communication. When people in the UK who had never discussed their end of life wishes were asked why [24], the most common reason given was that death seemed a long way off. In research with elderly care home residents [25] and kidney dialysis patients [26], many felt they were too busy with day to day life to consider end of life wishes. Many people with COPD preferred to concentrate on staying alive than on planning for their death [27]. Lack of knowledge of the options may be an issue. In Ireland, 71% of people had not heard of an advance directive [23]. This may in part be related to the complex medical and legal terminology often used by professionals. Some people may also feel that they do not have genuine choices about end of life care; many care home residents in the UK thought that the decision on whether they could stay in place at the end of their life would be made by other people [25].

Some people find contemplating their own death upsetting or frightening [28]. Others find having these conversations with people close to them difficult. In research from the UK and USA, older people said they would like to talk to their families about their end of life wishes but their families did not wish to [13,29-32]. In the USA, older adults and their adult children talked of the problem of finding time to talk when families lived at a distance [30]. Talking about death has often been described as ‘taboo’ [33,34]. However, in a recent UK survey, 71% of people agreed that they felt comfortable talking about death with friends and relatives [22], although almost the same proportion said they thought most people in Britain felt uncomfortable talking about death.

Dying, death and bereavement are increasingly being recognised as public health issues [35-37] and the need for the ‘normalisation’ of death has been recognised [37,38] by policy makers. Listening events with older people in the UK revealed that people are willing to engage in discussion about end of life issues [29].

The purpose of this review is to establish current evidence for the effectiveness of public health interventions to encourage people within the general population to consider, and to discuss with those close to them, their preferences for end of life care or what they wish to happen after their death.

## Methods

### Inclusion criteria

Studies were included if they described and evaluated a community-based intervention designed either to encourage people to consider, and to discuss with those closest to them, their preferences for end of life care or what they would wish to happen after death, or to address known barriers to these discussions. Known barriers to discussions are described in the Background and include:

•Not considering the issue worth considering at the moment

•Lack of knowledge of the options available

•Fear or distress associated with thinking about death or dying

•Difficulty persuading significant others to participate in these conversations, or fear of upsetting others

Included studies had to report on at least one outcome relating to attitude or behaviour change in the target group, or perceptions of the intervention as reported by the target group. Direct observations by researchers or staff delivering the interventions were acceptable if quantified or supported by specific examples.

Studies were excluded if they included only people with a life-limiting illness; evaluated only interventions designed specifically to facilitate communication of end of life preferences between patients and healthcare staff; or were intended only to facilitate the completion of advance care planning documents.

### Search criteria and methods

An initial search was conducted using Scopus. The search terms used were:

(‘Dying’ OR ‘End of Life’) AND (‘Planning’ OR ‘Public Health’ OR ‘Health Promoting Palliative Care’ or ‘Health Promotion’ or ‘Discussion’ or ‘Talk’ or ‘Conversation’ or ‘Communication’)

Terms listed in article title, abstract or keyword

Dates Jan 1, 2000 to August 20, 2013.

Limit to Health Sciences and Social Sciences and Humanities

A Google search using the same search terms was used to identify books and websites that were not included within academic databases.

Following the application of inclusion criteria, academic experts working in the field of public health and palliative care were contacted and asked about any additional relevant published work which they knew about.

### Selection of included studies

All publications which appeared to cover a related topic were retrieved, read and the reference lists were scanned for further relevant publications. Selection of studies by application of the inclusion criteria was then undertaken by the first author.

### Data extraction and analysis

Each study was summarised by study intervention, target group, research or evaluation methods, and findings. Findings were categorised as either:

•Primary outcomes, relating to evidence of encouraging discussions between participating targets and people close to them, or;

•Secondary outcomes relating either to addressing known barriers to discussion or to intermediate outcomes such as attendance at an event, evidence of engagement in a process, or participants’ ratings of the intervention.

The quality of the included studies was assessed using the system developed by Hawker and Payne [39] for reviews including studies using a diversity of methods (Appendix 1). Studies were scored on nine criteria, using the following scoring system: Good = 4; Fair = 3; Poor = 2; Very Poor = 1. Total scores were calculated for each study, where 9 = lowest possible (very poor) and 36 = highest possible (very good). Where a study was described in more than one paper, the best description available was used. Where a criterion was not relevant to the study, for example, ethical approval for an evaluation, the study was scored as ‘Good’ for that criterion.

Data extraction and analysis were undertaken by the first author and last author and reviewed by all authors. No attempt was made to combine study results, because the small number of studies and wide range of interventions reported made this inappropriate. All authors contributed to the interpretation of findings.

This review is reported according to PRISMA guidelines.

## Results

### Search results

The Scopus search returned 5,743 citations. The Google search revealed around 636 millions results, of which the first 40 pages were screened. The experts contacted were not aware of any additional relevant studies. In many cases it was difficult to determine the content of an article from its title; as a result over 400 abstracts were scanned, and over 100 full-text articles and two books were retrieved. All potentially relevant articles were either written in English or had an abstract in English. The most common reasons for exclusion of studies were that they were not intervention studies, or that the target group were people already known to have a life-limiting illness, usually involving advance care planning with healthcare staff. A book chapter describing various projects undertaken by a London hospice to engage local communities in discussion about life, death and bereavement was excluded because it did not provide enough detail for us to be able to extract data relating to specific interventions and outcomes [40].

**Figure 1:**
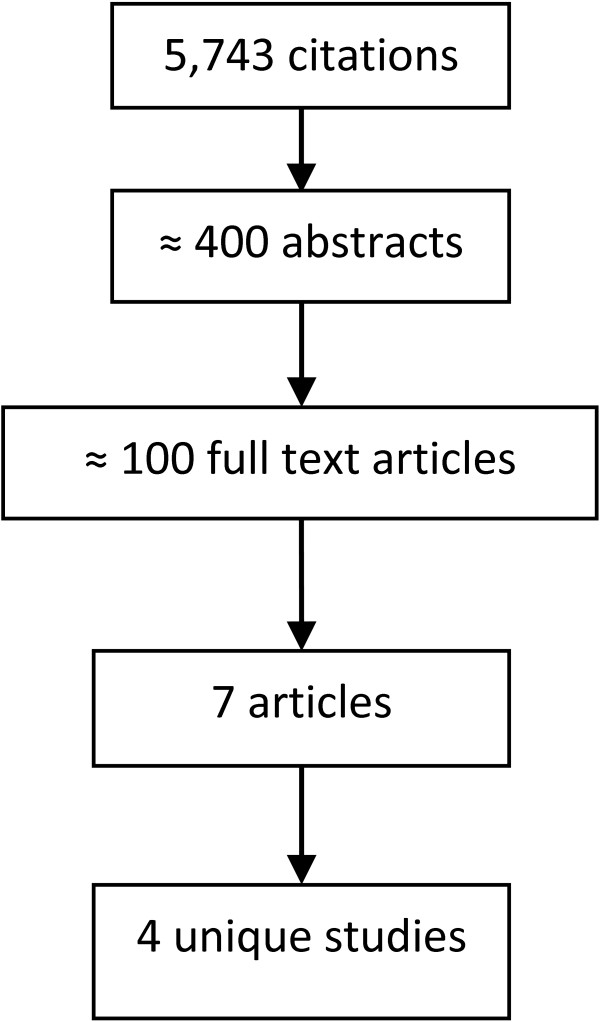
Flowchart illustrating selection of included studies from Scopus search.

From the retrieved studies, five studies, described within seven journal articles and one book chapter, were included. Four were identified by the search and one (a book chapter) was identified through the Google search. The number of studies identified at each stage of the Scopus search and selection procedure is summarised in Figure 1.

### Characteristics of included studies

Four of the included studies were undertaken in the UK and one was undertaken in Japan. The aims, interventions and research or evaluation methods of the included studies varied widely. The studies, and the reasons for their inclusion, are summarised in Table 1.

**Table 1:** Summary of included studies

**Study ID and location**	**Aim of intervention**	**Intervention methods**	**Reason for inclusion**	**Evaluation methods**	**Main findings**
**Miyashita et al.**[45,46] Fukashima, Japan	To raise awareness among the general public of the possibility of receiving appropriate support to enable dying at home	One-hour lectures delivered by a physician at a series of public meetings. Lectures covered treatment options and resources available to people who were dying at home.	Addressed known barrier: lack of knowledge of the options available	Quantitative questionnaire survey of attendees before, immediately after and six months after the lecture.	99% of attendees reported previously discussing end of life concerns with family.
		Target group: self selected by open advertisement		607 people attended, 595 completed questionnaire before and immediately after the lecture, 424 at all three time-points.	95% said the lectures would help in the future.
					10% of those completed all three questionnaires stated that home death was possible before the lecture, rising to 37% immediately following the lecture but falling back to 12% after 6 months.

**Seymour and Clarke et al.**[41-43] North of England, UK	To engage and educate older people on end of life planning options and processes; as both educators and educated.	Collaboration between academic staff and older people from voluntary agencies to develop an information booklet and peer education programme designed to facilitate peer to peer discussions.	Addressed known barriers: lack of knowledge of the options available and lack of opportunity for discussion	Questionnaires (n = 12) and telephone (n = 8) interviews of people who attended the workshops	In questionnaires and interviews workshop participants reported the booklet and opportunity to discuss issues with their peers to be worthwhile and useful.
		Target group: people over the age of 65		Focus groups of the peer educators	In focus groups the peer educators said they found the experience enjoyable and rewarding but most did not feel confident enough to lead a workshop themselves.
**Hartley**[47] London, UK	To change perceptions of death and dying among school children and their families	Project which brought school pupils and hospice users together to answer children’s questions and work on an arts project of their choice, and which ends with a presentation of the project to parents.	Addressed known barriers: fear of death and dying, lack of knowledge of the options available	Ongoing evaluation: questionnaires, including space for free text, completed by all participants.	Qualitative analysis of free text responses identified ‘normalising death and dying’ as a major theme for the children involved.
		Target group: school pupils, teachers, parents and hospice users		The exact sample size was unstated, although by the point of analysis, as the project had been run over 40 times.	Example: “....we thought they’d all be miserable and depressed.......but it was just like being with your friends.....we laughed and cried and sometimes felt afraid, normal things....” (16 year old pupil)
**Sanders et al.**[44] Various locations, UK	To educate people with long term health condition about end of life care planning	Short learning module within a much wider generic ‘expert patient’ course designed to teach people how to better manage any long term health condition	Addressed known barrier: lack of knowledge of the options available	Qualitative analysis of interviews with people who attended the course, using purposive sampling for maximum variation and across all areas of England 2(n=31).	The majority of participants thought the advance care planning module was inappropriate in the context it was introduced. Some people, who had recently been bereaved, felt distressed during the session.
		Target group: People diagnosed any long term health condition, who self-referred onto the course. Participants were not made aware of the end of life care planning module in advance of the course.			
**Hickey et al.**[32]Essex, UK	To educate and engage the public in discussing end of life issues	Well-advertised public information roadshows were held in two busy town centres. People who attended were invited to complete an end of life planning questionnaire, with support available to respond to any queries arising.	Aimed directly to encourage people to consider and discussed their end of life preferences; also addressed the known barrier of lack of knowledge of the options available.	Record keeping and observation of staff who delivered the intervention	The events were reported to be well attended by people of all ages. More than 450 people completed questionnaires, 70% of them female.
		Target group: members of the public attending a town centre outdoor event with an end of life theme			Staff observed the process of completing questionnaires help start discussions about end of life wishes among people who completed the activity together. The authors also reported that many people also accessed information, support and referral as a result.

Only one study [32] evaluated an intervention designed to directly influence people to discuss their end of life preferences with those closest to them and to evaluate this effect. This was a public information roadshow with an opportunity for people attending to complete a questionnaire together. Two further studies [41-44] were designed primarily to increase knowledge of end of life planning, although the interventions themselves included opportunities for group discussion with peers. One study used public lectures to raise awareness of options for end of life care [45,46] and another was an arts-based project designed to educate school pupils about the work of a hospice and the realities of dying [47].

The research methods used to evaluate the interventions included qualitative interviews; qualitative analysis of free text comments on questionnaires; mixed methods of questionnaires, telephone interviews and focus groups; a quantitative ‘before and after’ questionnaire survey; and direct observation by the people delivering the interventions.

### Quality of included studies

In general, the quality of included studies was assessed to be good, with quality scores ranging from 29 to 36 (Table 2). However, this hides significant weaknesses in the studies’ methodologies as they relate to the review question. Scores were boosted by our decision to assign maximum scores for criteria which were not relevant for particular studies. One of the studies in particular [32] was a simple descriptive observational study and many of the items included in the standard quality assessment tool used were not relevant. We also scored each study as ‘good’ in terms of usefulness because of the scarcity of other evidence in the field. The majority of included studies were written up well, which boosted their score using the system selected, which assesses quality of writing as much as quality of research design and conduct. The studies also to tended to score highly for methodology because they used an appropriate and well described method; however, methods tended to be limited in breadth and scope, and most were designed primarily to answer a slightly different question to that of the review. For example, one study [45,46] used a purely quantitative questionnaire survey, meaning that more subtle or unexpected effects may not have been captured. Another used open text responses from questionnaires administered immediately following an intervention [47], therefore limiting the study to people’s immediate observations, and those which could be written in a small space.

**Table 2:** Quality assessment of included studies

**Study ID**	**Aspect**	**Assessment**	**Score**	**Comments**
**Miyashita et al.**[45]	Abstract and title	Good	4	
	Introduction and Aims	Good	4	
	Method and data	Good	4	Questionnaires not presented but described in detail
	Sampling	Good	4	
	Data analysis	Good	4	
	Ethics and bias	Good	4	
	Findings/results	Good	4	
	Transferability/generalisability	Good	4	
	Implications and usefulness	Good	4	
	**Total**		**36**	
**Seymour and Clarke et al.**[41]	Abstract and title	Good	4	
	Introduction and Aims	Good	4	
	Method and data	Good	4	
	Sampling	Good	4	
	Data analysis	Good	4	
	Ethics and bias	Fair	3	Ethical approval not relevant, evaluation study. Presents discussion of limitations.
	Findings/results	Good	4	
	Transferability/generalisability	Good	4	
	Implications and usefulness	Good	4	
	**Total**		**35**	
**Hartley 2012**	Abstract and title	Poor	1	Abstract not normally expected in the format of a book chapter
	Introduction and Aims	Good	4	
	Method and data	Fair	3	Questionnaires not presented
	Sampling	Good	4	
	Data analysis	Fair	3	Described simply as ‘content analysis’
	Ethics and bias	Fair	3	Ethical approval not relevant, evaluation study
	Findings/results	Good	4	
	Transferability/generalisability	Good	4	
	Implications and usefulness	Good	4	
	**Total**		**30**	
**Sanders et al.**[44]	Abstract and title	Good	4	
	Introduction and Aims	Good	4	
	Method and data	Good	4	
	Sampling	Good	4	
	Data analysis	Good	4	
	Ethics and bias	Fair	3	Ethical approval not relevant, evaluation study
	Findings/results	Good	4	
	Transferability/generalisability	Good	4	
	Implications and usefulness	Good	4	Focussed on acceptability rather than outcomes
	**Total**		**35**	
**Hickey**[32]	Abstract and title	Fair	3	
	Introduction and Aims	Fair	3	
	Method and data	Fair	3	Simple observations reported, but not clear who did the observing
	Sampling	Good	4	Not applicable, descriptive observational study
	Data analysis	Fair	3	Not applicable, descriptive observational study
	Ethics and bias	Fair	3	Intervention and evaluation methods had few ethical and bias issues
	Findings/results	Poor	2	Did not quantify the numbers of people who engaged in discussion and provided only one example; however this was not the main topic of the paper
	Transferability/generalisability	Good	4	
	Implications and usefulness	Good	4	
	**Total**		**29**	

Results are presented separately by primary and secondary outcomes.

### Primary outcomes

Only one study reported on the primary outcome of the review. Hickey [32] reported that many people who completed an informal questionnaire survey together at a public information road show had engaged in discussion together about their end of life wishes. This was observed by people who were facilitating the questionnaire. They gave the following example: a married couple who had never spoken about their end of life preferences agreed to complete a questionnaire supported by a professional with palliative care experience. Both were surprised at the wishes of the other and continued in conversation with one another about these issues, with no need for further facilitation.

### Secondary outcomes

#### Engagement, attendance, and participant views

Hickey 2013 also reported that the public information ‘roadshows’, which had been well advertised and were located in two busy town centres in the South East of England, were well attended by people of all ages and more than 450 people participated in a facilitated questionnaire survey, approximately 70% of them female [32]. It was also reported that many people were able to access information, support and referral as a result of completing the questionnaire, although this observation was not quantified.

An action research study to pilot an older person’s peer education project in the North of England [41-43] demonstrated that it was feasible to develop a high-quality educational booklet on end of life planning in collaboration between academic staff and older people from voluntary agencies. The booklet covered end of life choices and planning, ethical issues, caring and coping, and loss and bereavement. After training, older volunteers also helped to facilitate a series of three end of life planning workshops for peers, which were each attended by six to eight older people. In structured questionnaires (n = 12) and semi-structured telephone interviews (n = 8), older people attending the workshops said they considered the educational booklet provided, and the opportunity to discuss issues with their peers, to be worthwhile and useful. A focus group with peer educators (volunteers) helping to design and deliver the project revealed that they found the experience rewarding, and that they thought they had learned a lot, but most did not feel confident enough to lead the workshops and preferred the role of assistant.

A London project, bringing together hospice users and school pupils to work together on an arts project to present to parents (Hartley 2012), reported being successfully run over 40 times, with a range of different schools and age groups. It was observed that children asked questions and hospice users talked freely about the experience of illness and dying. Most participants also completed an evaluation questionnaire at the end of the particular project they were involved in. In free-text responses, participants (children, parents and hospice users) reported various positive personal outcomes. For example a ten year old child wrote ‘…my grandmother died at the hospice and I wasn’t allowed to go…I enjoyed seeing that it was OK really’, a parent wrote ‘I’ve lived in this area all my life and have been too afraid to come into the building…is it possible to volunteer some of my time to continue to help?’ and a hospice user wrote ‘I always felt nervous talking to my children about what was happening to me – couldn’t find the words and didn’t want to upset them…watching people talk to each other here gives me the confidence to talk to my own family’.

A public lecture programme in Japan, on the topic of home-based end of life care [45,46] was attended by 607 people, although the lectures were combined with regional public meetings on other topics. The mean age of attendees was 66 years, 67% were female, and 84% reported excellent or good health. Most (99%) reported having already had discussions of end of life concerns with family. Of 595 people who attended; 95% said it was interesting, 96% said it was easy to understand, 95% said it would be of help in the future and 94% said it provided the opportunity to consider end of life medical treatment.

In a qualitative interview study of people in the UK who had attended an ‘Expert Patients’ course on self-management of a long term illness [44], the majority said that the subject of advance care planning was inappropriate in the context it was introduced. Some, who had recently been bereaved, were distressed and others felt that it was out of context with the course, which was about managing their health condition in a positive way. Others thought that there was not enough support available to deal with the sensitive issues raised, or that there was not enough time to discuss the issues in sufficient detail. Information materials for the Expert Patient’s course did not make any reference to the module of advance care planning, and therefore participants were not expecting it.

#### Normalisation of death

Content analysis of questionnaires completed by participants in the hospice-schools arts programme previously described identified four major themes: changing ideas and attitudes towards hospices (pupils), normalising death and dying (pupils); enjoyment that patients got from acting as educators (patients), and creating a relationship between the hospice and community (pupils and parents). Quotes included: “....we thought they’d all be miserable and depressed.......but it was just like being with your friends.....we laughed and cried and sometimes felt afraid, normal things....” (16 year old pupil)

#### Belief in the possibility of dying at home

In a questionnaire survey of 595 people attending the public lectures about home-based care in Japan, prior to the lecture, 9% of participants stated that home death was possible, 53% said it was impossible and 33% were unsure. Immediately after the lecture, 34% stated that home death was possible, 27% said it was impossible and 32% were unsure. This represented a significant change from ‘impossible’ to ‘possible’ (P = 0.001). Of these 595 participants, 424 also completed a questionnaire six months after the lecture. In this sample, 10% stated that home death was possible before the lecture, this rose to 37% immediately after the lecture but after six months later it fell to 12%. The difference between baseline and last follow up was not statistically significant (P = 0.12).

## Discussion

To our knowledge this is the first systematic review on this topic. We identified only five studies which met our inclusion criteria, despite a huge search and also speaking to key people in the field to ensure relevant papers had not been omitted. It is possible that we missed other published studies, despite our extensive search, as articles may not have been indexed as we expected. We also limited our initial search to Scopus and Google and to studies published in 2000 or later. This was because we expected this to be a relatively recent field of study, and needed to limit the number of irrelevant citations in a search which already had a low specificity. However, the reference lists of identified studies were scanned for earlier studies and findings do suggest a genuine scarcity of research evidence in this area.

In addition to the small number of studies, the studies available presented fairly limited evidence for what can be effective in encouraging people who are well to discuss their end of life wishes with those closest to them. The majority of studies aimed primarily to answer slightly different questions, and some seemed to have been severely limited by the funding available. Only one study reported on the primary outcome of this review, and this was quite low quality, almost anecdotal evidence, based on observations made during an intervention. Two other studies reported observations that interventions appeared to help to facilitate conversations about end of life planning (older people) or death and dying in general (school pupils), but these conversations occurred among peers rather than among close family and friends. The methods employed by studies are also often quite limited in scope, for example, most studies used only very short-term follow-up, while the true effects of an intervention may take some time to be felt. Studies which used only quantitative methods or only free text responses from self-administered questionnaire may not have captured all of the subtleties of effects, while one study which used in-depth interviews concentrated on the acceptability of an intervention rather than its outcomes.

Despite the paucity of evidence, some useful findings have been identified. In one study, couples attending a public information ‘roadshow’ event, who were engaged in completing an informal end of life planning questionnaire survey together, were observed to often become involved in discussions of end of life wishes between themselves, sometimes for the first time. Although this finding is not quantified, and comes from a relatively poor quality descriptive observational study, this evidence is direct and cannot be discounted.

Another intervention was shown to be successful in engaging older people in discussion about end of life planning with peers. Older volunteers were employed as peer educators alongside academic staff, resulting in a user-friendly end-of-life planning information booklet and an associated workshop that was valued by the participants. A project bringing together school children and hospice patients to work together on an arts project reported facilitating natural conversations between school pupils and hospice users, in the process helping to normalise death and dying for children and young people. Normalising death may help allay some of the fears that can make talking about death and dying more difficult, and hence projects like this may facilitate discussions about end of life in the long term.

A less successful intervention in engaging people and facilitating discussion included an end of life care planning module within an ‘expert patient’ education programme, designed to help patients to self-manage conditions that were not necessarily life-limiting. The majority of participants felt that the topic was inappropriate or distressing, and did not wish to discuss it.

An intervention using public lectures to try to change beliefs in the possibilities for end of life care had limited success. The lectures attracted mainly people who had already discussed their end of life preferences with family, and did not significantly change beliefs about the possibilities for end of life care beyond the very short term.

We know anecdotally, and through our search and reading, of several recent and ongoing projects in the UK and worldwide which include within their aims encouraging people within the general population to consider their end of life preferences or discuss these more openly with those close to them. This suggests either that projects are not being formally evaluated for publication, or that this is still a relatively new area of practice and research, and that evaluations have not yet been conducted. It seems most likely explained by a combination of these two factors. In order to advance knowledge in this subject area, it is important that people evaluate and, if possible, publish the evaluations of any projects they are undertaking in this area. More research is also needed.

## Conclusions

There is currently very limited research evidence regarding the effectiveness of different interventions to encourage people who are currently well to consider and discuss their end of life preferences with the people closest to them. Available evidence suggests that passive lectures or presentations are unlikely to be as effective as participatory approaches. It has also highlighted the importance of finding an appropriate context for interventions and of sensitivity to those who may not wish to engage in discussion about end of life issues at the time.

It may be difficult to assess the effectiveness of many interventions, which have subtle and long-term aims; this review has illustrated the importance of medium and long term follow-up. However we would encourage all those involved in the increasing number of public health approaches to palliative care projects internationally to evaluate their work to allow the body of evidence on this increasingly important area to be collated and used to inform wider discussion and further developments.

## Appendix 1

Quality Assessment Criteria

1. Abstract and title: Did they provide a clear description of the study?

***Good:*** Structured abstract with full information and clear title.

***Fair:*** Abstract with most of the information.

***Poor:*** Inadequate abstract.

***Very Poor:*** No abstract.

2. Introduction and aims: Was there a good background and clear statement of the aims of the research?

***Good:*** Full but concise background to discussion/study containing up-to date literature review and highlighting gaps in knowledge.

Clear statement of aim AND objectives including research questions.

***Fair***: Some background and literature review. Research questions outlined.

***Poor:*** Some background but no aim/objectives/questions, OR Aims/objectives but inadequate background.

***Very Poor:*** No mention of aims/objectives. No background or literature review.

3. Method and data: Is the method appropriate and clearly explained?

***Good:*** Method is appropriate and described clearly (e.g., questionnaires included).

Clear details of the data collection and recording.

***Fair:*** Method appropriate, description could be better.

Data described.

***Poor:*** Questionable whether method is appropriate.

Method described inadequately.

Little description of data.

***Very Poor:*** No mention of method, AND/OR Method inappropriate, AND/OR No details of data.

4. Sampling: Was the sampling strategy appropriate to address the aims?

***Good:*** Details (age/gender/race/context) of who was studied and how they were recruited.

Why this group was targeted.

The sample size was justified for the study.

Response rates shown and explained.

***Fair:*** Sample size justified.

Most information given, but some missing.

***Poor:*** Sampling mentioned but few descriptive details.

***Very Poor:*** No details of sample.

5. Data analysis: Was the description of the data analysis sufficiently rigorous?

***Good:*** Clear description of how analysis was done.

Qualitative studies: Description of how themes derived/ respondent validation or triangulation.

Quantitative studies: Reasons for tests selected hypothesis driven/ numbers add up/statistical significance discussed.

***Fair:*** Descriptive discussion of analysis.

***Poor:*** Minimal details about analysis.

***Very Poor:*** No discussion of analysis.

6. Ethics and bias: Have ethical issues been addressed, and what has necessary ethical approval gained?

Has the relationship between researchers and participants been adequately considered?

***Good Ethics:*** Where necessary issues of confidentiality, sensitivity, and consent were addressed.

***Good Bias:*** Researcher was reflexive and/or aware of own bias.

***Fair:*** These issues were acknowledged.

***Poor:*** Brief mention of issues.

***Very Poor:*** No mention of issues.

7. Results: Is there a clear statement of the findings?

***Good:*** Findings explicit, easy to understand, and in logical progression.

Tables, if present, are explained in text.

Results relate directly to aims.

Sufficient data are presented to support findings.

***Fair:*** Findings mentioned but more explanation could be given.

Data presented relate directly to results.

***Poor:*** Findings presented haphazardly, not explained, and do not progress logically from results.

***Very Poor:*** Findings not mentioned or do not relate to aims.

8. Transferability or generalizability: Are the findings of this study transferable (generalizable) to a wider population?

***Good:*** Context and setting of the study is described sufficiently to allow comparison with other contexts and settings, plus high score in Question 4 (sampling).

***Fair:*** Some context and setting described, but more needed to replicate or compare the study with others, PLUS fair score or higher in Question 4.

***Poor:*** Minimal description of context/setting.

***Very Poor:*** No description of context/setting.

9. Implications and usefulness: How important are these findings to policy and practice?

***Good:*** Contributes something new and/or different in terms of understanding/insight or perspective.

Suggests ideas for further research.

Suggests implications for policy and/or practice.

***Fair:*** Two of the above (state what

***Poor:*** Only one of the above.

***Very Poor:*** None of the above.

## Competing interests

The authors declare that they have no competing interests.

## Authors’ contributions

KA undertook the searches, selected studies for inclusion, summarised the included studies, assessed the quality of the included studies and drafted the manuscript. PB and SH contributed to the design of the review and interpretation of results. MLW conceived the idea for the review, and contributed to its design, assessment of quality of included studies, and interpretation of the results. All authors contributed to the writing of the final manuscript and agreed its contents for publication.

## Authors’ information

KA is currently a full-time PhD student at the University of Liverpool with previous experience of conducting systematic reviews. PB is a Lecturer in Sociology of Health and Illness within Liverpool Medical School and is involved in research around diabetes, A&E presentations, and Opiate Substitution Therapy. SH is the matron at St Luke’s Cheshire Hospice. She has worked in specialist palliative care for 22 years, and her interests include the role of specialist palliative care in changing public knowledge and behaviour in relation to death and loss. MLW is a Professor at the University of Liverpool, undertaking psychosocial research in palliative care and she is also an Honorary Consultant in Palliative Medicine undertaking clinical outpatient work.

## Pre-publication history

The pre-publication history for this paper can be accessed here:

http://www.biomedcentral.com/1472-684X/12/40/prepub
